# New Insight into the Chloroacetanilide Herbicide Degradation Mechanism through a Nucleophilic Attack of Hydrogen Sulfide

**DOI:** 10.3390/ijms19102864

**Published:** 2018-09-21

**Authors:** José R. Mora, Cristian Cervantes, Edgar Marquez

**Affiliations:** 1 Instituto de Simulación Computacional (ISC-USFQ), Universidad San Francisco de Quito, Diego de Robles y Vía Interoceánica, Quito 17-1200-841, Ecuador; jrmora@usfq.edu.ec (J.R.M.); ccervantesv@estud.usfq.edu.ec (C.C.); 2 Grupo de Química Computacional y Teórica (QCT-USFQ), Departamento de Ingeniería Química, Universidad San Francisco de Quito, Diego de Robles y Vía Interoceánica, Quito 17-1200-841, Ecuador; 3 Departamento de Química y Biología, Facultad de Ciencias, Universidad del Norte, Km 5, Vía Puerto Colombia, Barranquilla 081007, Colombia

**Keywords:** density functional theory, computational modeling, transition state, herbicides, reaction mechanism

## Abstract

The nucleophilic attack of hydrogen sulfide (HS^−^) on six different chloroacetanilide herbicides was evaluated theoretically using the dispersion-corrected hybrid functional wB97XD and the 6-311++G(2d,2p) Pople basis sets. The six evaluated substrates were propachlor (A), alachlor (B), metolachlor (C), tioacetanilide (D), β-anilide (E), and methylene (F). Three possible mechanisms were considered: (a) bimolecular nucleophilic substitution (S_N_^2^) reaction mechanism, (b) oxygen assistance, and (c) nitrogen assistance. Mechanisms based on O- and N-assistance were discarded due to a very high activation barrier in comparison with the corresponding S_N_^2^ mechanism, with the exception of compound F. The N-assistance mechanism for compound F had a free activation energy of 23.52 kcal/mol, which was close to the value for the corresponding S_N_^2^ mechanism (23.94 kcal/mol), as these two mechanisms could occur in parallel reactions with almost 50% of each one. In compounds A to D, an important electron-withdrawing effect of the C=O and C=S groups was seen, and consequently, the activation free energies in these S_N_^2^ reactions were smaller, with a value of approximately 18 kcal/mol. Instead, compounds E and F, which have a CH_2_ group in the β-position, presented a higher activation free energy (≈22 kcal/mol). Good agreement was found between experimental and theoretical values for all cases, and a reaction force analysis was performed on the intrinsic reaction coordinate profile in order to gain more details about the reaction mechanism. Finally, from the natural bond orbital (NBO) analysis, it was possible to evaluate the electronic reorganization through the reaction pathway where all the transition states were early in nature in the reaction coordinate (δBav < 50%); the transition states corresponding to compounds A to D turned out to be more synchronous than those for compounds E and F.

## 1. Introduction

Among the diversity of chemical compounds, pesticides constitute a heterogeneous category used specifically for the control of pests, plant diseases, and to eradicate the unwanted weeds. The synthesis and production of pesticides represent very important fields of industry. Currently, the use of pesticides remains the most effective process for the protection of plants from pests; moreover, pesticides contribute significantly to enhancing productivity and crop yields around the world [1].

Herbicides are a class of pesticides that stand out for their expanded use around the entire world. Four of them (acetochlor, alachlor, butachlor, and metolachlor) are widely used in the production of several economically important crops. The annual production of these four compounds is about 125 million pounds per year; just metolachlor alone has an annual production of 65 million pounds per year in the United States (US) [2].

Despite the importance of the production of herbicide, one aspect is still dramatic: the carcinogenic potential of chloroacetanilide herbicides. Alachlor, acetochlor, and metolachlor were characterized by the US Environmental Protection Agency as likely or possible carcinogenic compounds. However, the carcinogenic mechanism of chloroacetanilide compounds is still unclear, despite some experiments showing evidence that the carcinogenic properties relate to the herbicides’ ability to react with DNA through nucleophilic reactions [3,4].

Another important characteristic of chloroacetanilide herbicides is their resistance to natural degradation in various environments. Their water solubility and great persistence in the environment represent topics of greatest interest in the scientific community. Currently, several scientific researchers are trying not only to avoid pollution, but are also searching for methods that help mitigate the already polluted soil and water systems [4].

Even with the already known environmental persistence and the pollutant characteristic of chloroacetanilide herbicides, until now, there are few decontamination methods used to remove or degrade these compounds. Bioremediation, chemical oxidation, and physical methods are used without much success; therefore, it is imperative to search for new, safe, effective, and low-cost decontamination methods for chloroacetanilide herbicide pollution [4,5,6,7].

Recently, some reports suggested a high selectivity of chloroacetanilide herbicides in the reaction with inorganic compounds such as sulfur nucleophiles like HS^−^, S^2−^, and S_2_O_3_^2−^; moreover, several earlier works documented the great reactivity of chloroacetanilide compounds toward the thiolate group of glutathione (GSH) [8,9]. Considering the importance of GSH in the biologic activity of plants, this latter characteristic allows the widespread use of chloroacetanilide for crop production. In other words, the knowledge of the reaction mechanism for chloroacetanilide against sulfur nucleophiles has a pivotal role in understanding not only the action pathway of phytotoxicity, but it could also be an opportunity to design new chemical remediation methods [8].

Several experiments were performed in order to understand the reactivity of chloroacetanilide herbicides toward thiolate groups. Even though the nucleophilic displacement of chlorine by the thiolate group of glutathione (GSH) seems to overall follow second-order kinetics via an intermolecular bimolecular nucleophilic substitution (S_N_^2^) mechanism, some reported experimental results for similar compounds do not support this mechanism; e.g., propachlor obeys a second-order law, while methylene (analog of propachlor) obeys a first-order law [9,10]. Until now, the reaction mechanism of chlorine displacement to the sulfur nucleophile remains unclear, and it seems to be a more complex mechanism than a bimolecular nucleophilic substitution (S_N_^2^).

Some authors proposed different reaction pathways in order to explain the kinetic behavior of chloroacetanilide toward sulfur nucleophiles. Bordwell et al. [11] stated that the activation of chlorine atoms could proceed from the interactions of anilide moiety with the incipient electrophilic center. They argued the electronic interactions of carbonyl substituents activate alkyl halides toward S_N_^2^ reactions with strong nucleophiles, while the carbonyl moiety is able to deactivate the reactivity toward weaker nucleophiles such as amines. In contrast, a considerable increase in the electrophilicity characteristic of the CH_2_Cl group due to the α-carbonyl moiety’s electronic effects was suggested; consequently, the compounds are able to react toward softer nucleophiles such as GSH and other SH-containing moieties via an S_N_^2^ mechanism [11,12,13].

Arcelli et al. [14] explained the reactivity of chloroacetanilide herbicides via anchimeric assistance provided by the ether oxygen. However, if this anchimeric assistance were the only factor responsible of reactivity, it would be expected that chloroacetanilides such as alachlor and metolachlor (which have *N*-alkoxyalkyl side chains) would be more reactive than propachlor (with an *N*-alkyl substituent). In fact, there is important evidence for this statement: propachlor is more reactive toward sulfur nucleophiles than alachlor and metolachlor; consequently, this result led us to invoke new alternative explanations.

Due to the uncertainty found with respect to the reactivity of chloroacetanilide with sulfur nucleophiles, it is necessary to explore, in more detail, the reaction mechanism of these substrates. In this sense, computational methods could be a helpful tool for this goal.

This work seeks to explore chloroacetanilide’s reactivity with HS^−^ nucleophiles in order to propose a reasonable mechanistic interpretation based on theoretical calculations. With this aim, the potential energy surfaces were examined using the density functional theory (DFT) level, and the results were analyzed and compared with the experimental data. The minimum energy structure of reactants, the transition state, and the product were calculated, taking into account all possible reaction mechanisms suggested in the literature.

The processes of rupture and bond formations were studied using natural bond orbital calculations (NBO). In addition, intrinsic coordinate path reactions (IRC) and reaction force profiles were used to gain more insight into the reaction pathway.

## 2. Results and Discussion

**Scheme 1:**
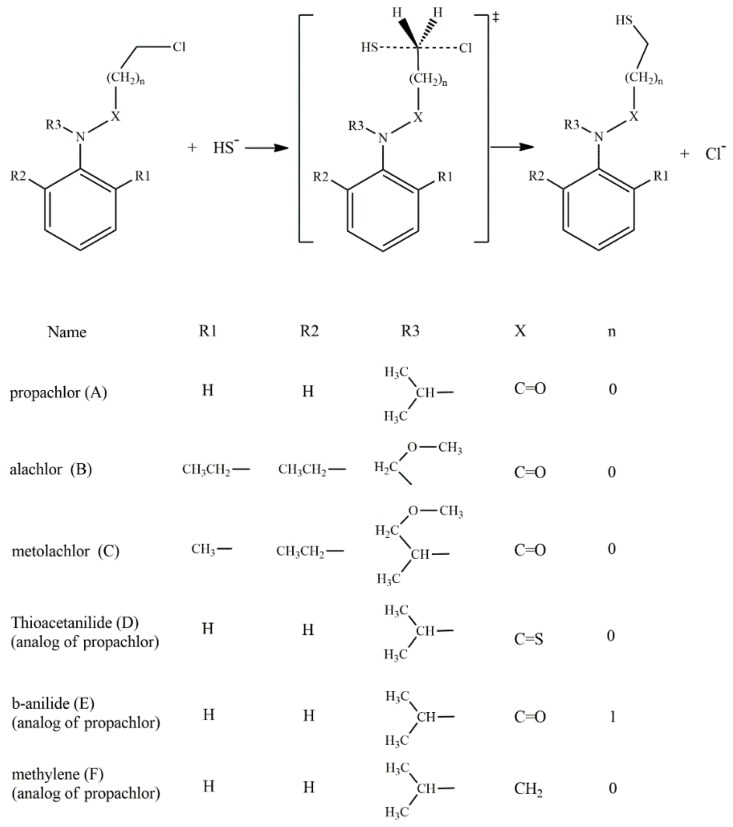
S_N_^2^ reaction mechanisms for the six compounds: A to F.

**Scheme 2:**
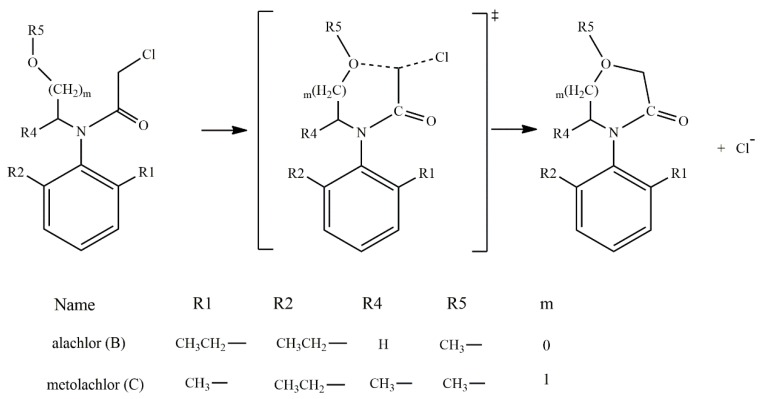
Oxygen anchimeric assistance for compounds B and C.

**Scheme 3:**
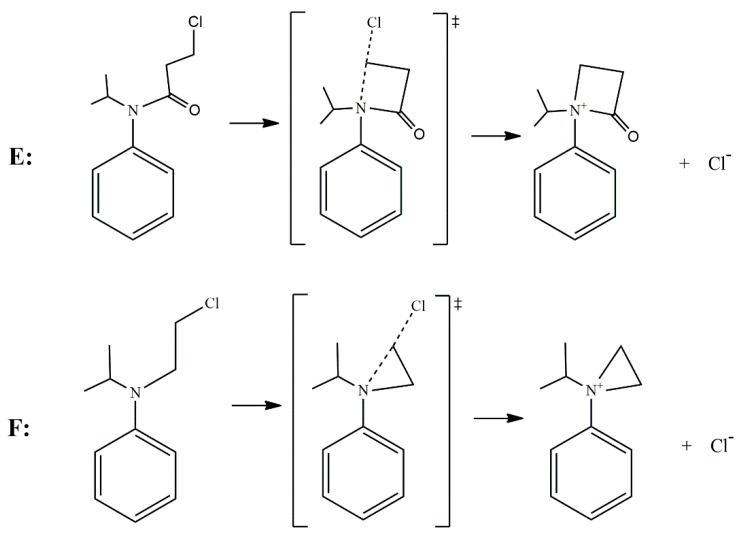
Nitrogen anchimeric assistance for compounds E and F.

According to the reported products for the reaction between HS^−^ and chloroacetanilide herbicides [15], and based on the structure of each substrate, three possible mechanisms can be suggested. The main mechanism is the well-known S_N_^2^ displacement (Scheme 1), which is proposed for all compounds. In the case of compounds B and C, the halogen elimination carried out by an anchimeric assistance of the oxygen atom can be considered (Scheme 2). For compounds E and F, due to the length of the carbon chain in the first case, and the lack of the carbonyl group in the second, there is the possibility of an anchimeric assistance by the nitrogen atom in the chloride elimination (Scheme 3).

### 2.1. Thermodynamic Parameters

The thermodynamic parameters for the ten reaction mechanisms described above are included in Table 1. These results are compared with the experimental values, and good agreement is found among all of them. The S_N_^2^ mechanism is suggested as the most plausible path for these reactions, with the exception of compound F.

The results reported in Table 1 show a good agreement between experimental and theoretical reactivity trends. In all cases where the oxygen anchimeric assistance was considered, higher values on the reaction barriers were found; therefore, this possibility can be discarded for those particular substrates. With respect to the nitrogen-assistance mechanism, for the case of compound F, it is also a favored mechanism, which is in agreement with the previously experimental evidence commented about these compounds [15]. Interestingly, the values of free energy of activation for the S_N_^2^ and N-assistance mechanism are very close, which suggest that these reactions occur in parallel with almost 50% of each case. Taking into account that the values of activation free energy of these two mechanisms are similar, these two mechanisms can be differentiated only by considering the changes in activation entropy, where the N-assistance mechanism present a small negative value (∆S^‡^ = −0.23) in consonance with a unimolecular process, while the S_N_^2^ mechanism presents a high negative activation entropy value (∆S^‡^ = −21.36) due to its bimolecular nature (loss in translational degree of freedom).

For the case of compound E, a small positive entropy value (4.97) is reported in the experimental work, which suggests a unimolecular process involving an anchimeric assistance through the nitrogen atom, as depicted in Scheme 3, where a possible four-membered ring can be generated as an intermediate. The theoretical thermodynamic parameters (∆G^‡^, ∆H^‡^, and ∆S^‡^) obtained for this possible mechanism were 41.45 kcal/mol, 41.38 kcal/mol, and −0.22 cal/molK, respectively. Clearly, the activation free energy for this N-assistance in compound E is higher than that corresponding to the S_N_^2^ mechanism; therefore, the assistance can be discarded.

**Figure 1:**
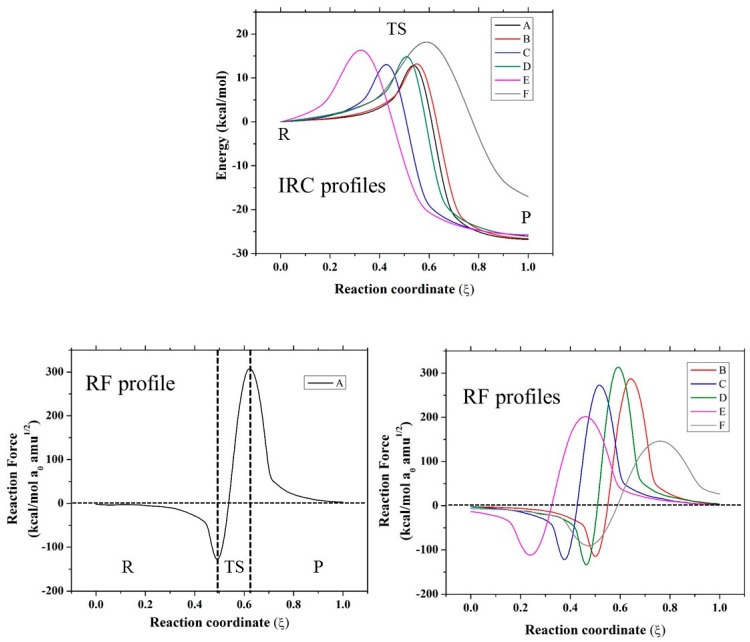
Intrinsic coordinate path reactions (IRC) and reaction force (RF) profiles of S_N_^2^ mechanism for all evaluated compounds.

**Figure 2:**
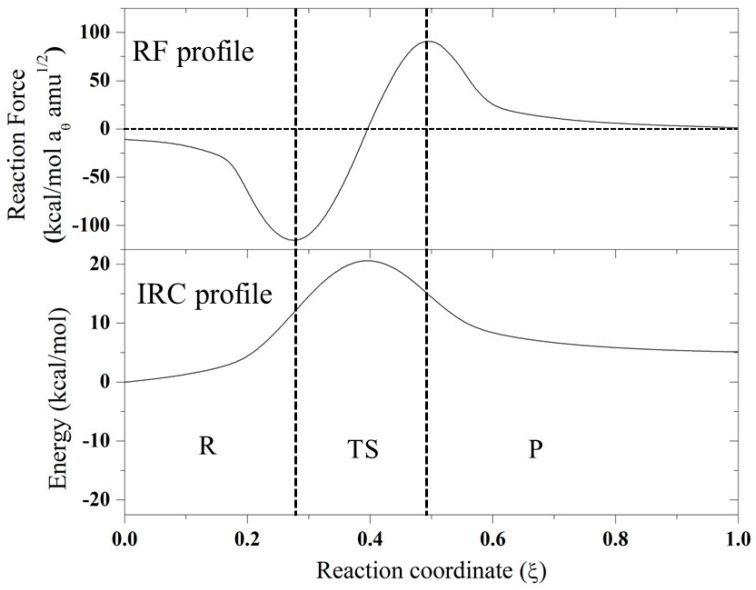
IRC and RF profile for the N-assistance mechanism for compound F.

Based on these results, for further analysis, the S_N_^2^ mechanism for compounds A–F and the anchimeric assistance for compound F were considered. In this sense, in Figure 1 and Figure 2, the IRC profiles for all the reactions studied in the present work are presented. Evidently, all the reactions are exothermic except for the case of the assistance of a nitrogen atom, which is endothermic. Compounds A, B, and C have similar values for the theoretical and experimental activation thermodynamic parameters involved in the S_N_^2^ mechanism, which is expected because they have the same reaction center with a neighboring carbonyl group, with the rest of the molecule groups far away from the reaction center. In the case of compound D, an oxygen atom is changed by a sulfur atom, and a small effect is observed due to this change: a small increase in the activation enthalpy value accompanied by a decrease in the activation entropy; however, this effect is compensated for, and a similar free energy of activation with respect to compounds A–C is found. When compounds A–D were compared with compounds E and F, a considerable difference was found in the thermodynamic activation parameters. Clearly, the carbonyl group exerts an electro-withdrawing effect, which implies a more electrophilic carbon, and consequently, a small activation barrier of the process. On the other hand, compounds E and F have a CH_2_ in the beta position instead of a CO; therefore, these compounds have higher barriers. Based on these results, further analysis is centered on the S_N_^2^ mechanism for all compounds, and the only possible anchimeric assistance considered is the N-assistance found as favorable for compound F. In addition, in Figure 1 and Figure 2, the reaction force (RF) profiles for all the mentioned mechanisms obtained as described in the methodology section are reported.

From the RF profiles shown in Figure 1 and Figure 2, a clear partition between three regions can be seen: the reactant region (R), the transition state region (TS), and the product region (P). All the reactions are concerted in nature, and it was possible to obtain the four associated values of work done (W_1_–W_4_) based on the integration of each of these regions (Table 2). This value of work done (W_i_) was used to characterize the reaction in terms of structural rearrangements (W_1_) and electron reorganization (W_2_) from the reactant to transition state. Considering W_3_ and W_4_, the reaction energy (Er) can be also estimated. In all the reaction mechanisms evaluated, W_1_ > W_2_, suggesting that these reactions are principally dominated by structural rearrangement (~70%) involving the approximation of the nucleophile, which is correlated with the high entropy values found for these reactions in Table 1. W_2_ began gaining an important role for compounds E and F, which have a CH_2_ group instead of a CO neighbor to the reaction center. On the other hand, for the case of anchimeric N-assistance, the electronic reorganization is more important due to the unimolecular nature of this reaction. In order to characterize, in more detail, all the stationary points considered in this work, we describe the changes in geometric parameters involved in these reactions in the next section.

### 2.2. Geometric Parameters

**Figure 3:**
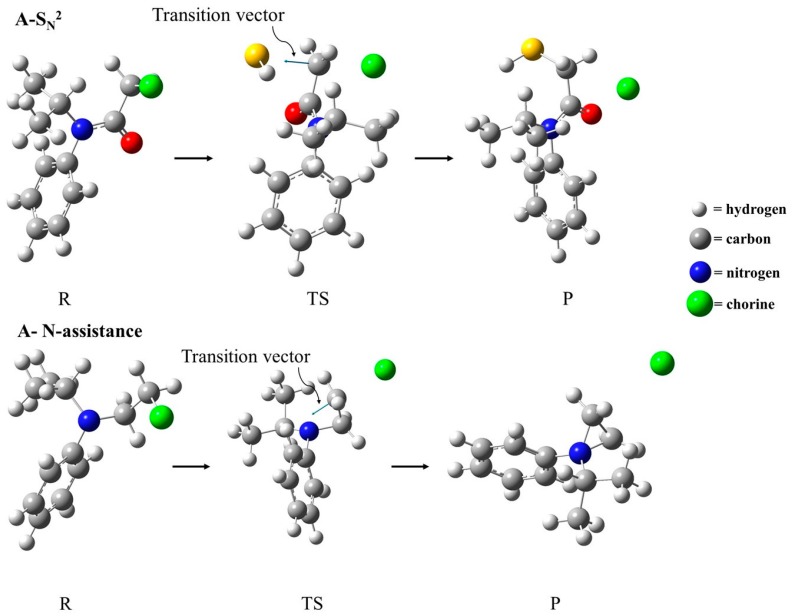
Optimized geometries of reactants (R), transition state (TS), and products (P) for the S_N_^2^ reaction mechanism of compound A and the N-assistance mechanism of compound F.

In terms of geometric parameters, similar changes in the S–C and C–Cl distances were found for compounds A–D. The S–C interatomic distance corresponding to the nucleophile approximation decreased from ~3.7 Å to ~2.5 Å, while the C–Cl dissociation increased from ~1.8 Å to ~2.2 Å (Table 3). Interestingly, the C–Cl bond in compounds E and F in the reactant, as well as in the transition state, were a little longer than the same bond in compounds A–D, which are in agreement with the electro-withdrawing effect of the CO group in compounds A–D, as discussed previously. With respect to the S–C–Cl angle, compounds A–D present more linear transition states (~170°) when compared to compounds E and F (~160°). The imaginary frequency values are associated with the transition vector (TV), shown in Figure 3 for the first-order transition state found as a saddle point. In an illustrative manner, Figure 3 depicts the optimized geometries for the reactant, transition state, and product for the compound A S_N_^2^ reaction mechanism, and the anchimeric N-assistance mechanism is described for compound F. The Cartesian coordinates for all the structures considered in this study are included in Table S1 in the Supplementary Materials.

### 2.3. Natural Bond Orbital (NBO) Analysis

The evolution on the electronic density through the reaction pathway plays an important role in the reaction mechanism, in order to gain information about the changes in the atom charges involved in the reaction mechanism for the different stationary points (reactants, transition states, and products). In this sense, the NBO charge changes from reactant to transition state, denoted by δQ*^x^* = (Q_TS_*^x^* − Q_R_*^x^*) for the corresponding *x* atom, are reported in Table 4. The net charges for each atom are included in Table S2 in the Supplementary Materials. Upon inspecting these results, it is evident that for compounds E and F, higher changes in the electronic density were found when compared to compounds A–D. The positive values of δQ^C^ and δQ^S^ suggest a decrease in electronic density in the carbon and sulfur atoms, while the chlorine atom acquired more electronic density with a negative value. Higher changes in the charge distribution were observed in the nucleophile and leaving group almost with the same magnitude in compounds A–D (more synchronic charge distribution); however, in compounds E and F, the charge distribution was less synchronic. On the other hand, in the N-assistance mechanism, a major decrease was observed in the electronic density of the carbon atom.

In order to gain more insight into the reaction mechanism, the Wiberg bond indexes for the bond involved in the transition state were determined, as listed in Table 5. The C–Cl bond represents the determining factor in the rate-determining step, which presents a bigger evolution through the reaction path with a δBi value of approximately 43%. The average value of δB_av_ < 50%, suggests that an early transition state is involved in the reaction mechanism, which is in agreement with the IRC profiles reported in Figure 1 and Figure 2. The synchronicity values suggest that the reactions for compounds A–D are more synchronous than the reactions for compounds E and F, which agrees with the discussion put forward in the NBO charge analysis above.

## 3. Computational Details

**Scheme 4:**
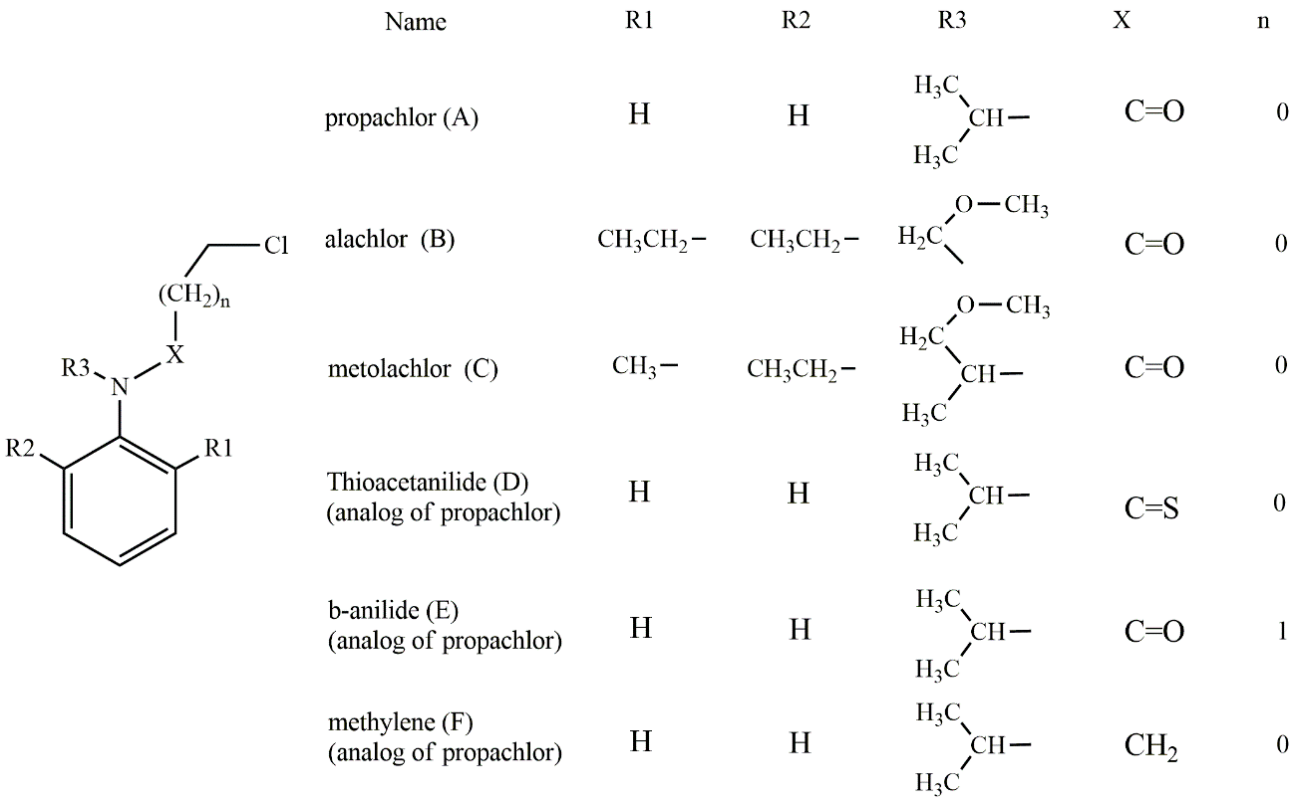
Chloroacetanilide herbicides evaluated in this work.

Based on the experimentally evidenced reactions [15], the nucleophilic attack of hydrogen sulfide (HS^−^) on chloroacetanilide herbicides, with the consequent displacement of a chloride atom, was studied in a total of six substrates: propachlor (A), alachlor (B), metolachlor (C), tioacetanilide (D), β-anilide (E), and methylene (F) (Scheme 4).

All these calculations were performed with the Gaussian16 suite [16] at the wB97XD/6-311++G(2d,2p) level. The long-range dispersion-corrected hybrid wB97XD functional [17,18,19] was shown to be adequate in the study of reaction mechanism [20,21]. The Pople basis set 6-311++G(2d,2p) was employed in order to adequately describe all the atom orbitals involved in the reaction, including the chloride atoms [22]. For the self-consistent field (SCF) calculations, the convergence criterion for the optimization process was set as default. To achieve the convergence in the density matrix, a value of 10^−9^ atomic units was required; the maximum displacement threshold value was 0.0018 Å and the maximum force threshold value was 0.00045 Hartree/Bohr. Reactants (R) and products (P) were characterized as minimum stationary points in the reaction coordinate. On the other hand, transition states (TS) were characterized as saddle points in the reaction path, with a unique negative eigenvalue on the force constant matrix, which was obtained by a frequency calculation on the optimized structures at 298.15 K [23]. With the frequency calculation, it is possible to obtain all the thermodynamic parameters, such as the zero-point energy (ZPE), absolute enthalpy (H), free energy (G), and entropy (S) values with the corresponding temperature correction, and the basis-set superposition error (BSSE) correction was considered for the transition state geometry [24]. A bimolecular nucleophilic substitution reaction (S_N_^2^) mechanism was considered for all the substrates, as well as some possible anchimeric assistance for oxygen or nitrogen atoms. The anchimeric O-assistance was considered for compounds B and C, and N-assistance for compounds E and F. The solvation effect was directly taken into account in the optimization process using the polarizable continuum model (PCM) with the solvation model density (SMD) proposed by Cramer and Truhlar, and water as a solvent [25,26].

Intrinsic reaction coordinate (IRC) profiles were constructed for the reaction mechanism departing from the transition state in the forward and reverse directions [27,28,29,30]. This method allows the verification of the connection between the reactant and product through the transition state. In order to further describe the corresponding mechanism, reaction force analysis (RF) was performed by taking the first derivative of the energy with respect to the reaction coordinate (F(ξ)=dE/dξ) [31,32,33,34]. This RF profile is valuable for describing the chemical changes along the reaction pathway in terms of electronic reorganization and structural rearrangements. These two contributions can be separately analyzed from the so-called works (Wi) obtained from the integration of each part on the reaction force profile.

The description of the electronic nature of all the stationary points was performed by means of natural bond orbital (NBO) analysis. In this sense, natural atomic charges (Q*^x^*) and bond orders (Wiberg indexes) were obtained for the optimized geometries of the reactants (R), transition state (TS), and product (P) ($B_{i}^{R},{\;B}_{i}^{\mathrm{TS}},B_{i}^{P}$). In Q*^x^*, the *x* represents an atom, and, for the evaluation of the charge changes through the reaction path, a δQ*^x^* = (Q_TS_*^x^* − Q_R_*^x^*) value was estimated for each *x* atom. With the Wiberg indexes, it was possible to estimate the evolution percent of each bond involved in the transition state using Equation (1):

(1) $$\;\%\mathrm{Ev}={\delta B}_{i}\;\times \;100=\left[\frac{B_{i}^{\mathrm{TS}}-B_{i}^{R}}{B_{i}^{P}-B_{i}^{R}}\right]\;\times \;100.$$

The evolution of each bond allows us to establish what the determining factor is in the transition state and how early or late the transition state is. These indexes are useful in determining how synchronous the transition state is in nature, as described by the synchronicity concept proposed by Moyano et al. [35], defined in Equation (2):

(2) $$\mathrm{Sy}=1-\frac{\left[\sum_{i=1}^{n}\frac{\left|{\delta B}_{i}-{\delta B}_{\mathrm{av}}\right|}{{\delta B}_{\mathrm{av}}}\right]}{2n-2}.$$

A value of zero for Sy advises a completely asynchronous process, and a value of 1 implies a synchronous process.

## 4. Conclusions

A mechanistic detailed study for the bimolecular nucleophilic substitution reaction of HS^−^ with chloroacetanilide compounds was performed using density functional theory at the wB97XD/6-311++G(2d,2p) level. An S_N_^2^ reaction mechanism was found as favorable for all compounds, with activation free energy values between 17 and 24 kcal/mol. Compounds A–D, which have the presence of a neighboring carbonyl group (C=O) and C=S, possess almost the same barrier (∆G^‡^ ≈ 19 kcal/mol). The C=O and C=S groups exert an electro-withdrawing effect favoring the nucleophilic attack, and consequently, a minor activation free energy was found in comparison with compounds E and F, which have a neighboring methylene group (CH_2_) in the same position. With respect to the reaction force analysis, in all cases, W_1_ > W_2_; W_1_ corresponds to the work done for geometric reorganization, corresponding to the formation of the transition state, and it represents about 70% of the total activation barrier for compounds A–D, and about 60% for compounds E and F. The charge evolution and bond-order analysis are in agreement with the reactivity trends described previously for the studied substrates: the presence of a carbonyl group reduces the electron density of the carbon atom in the CH_2_Cl group, which implies a smaller free activation energy. In compound F, the anchimeric N-assistance via a unimolecular process is also favored in almost 50% of cases, because the activation free energy of this mechanism is very close to the value found for the corresponding S_N_^2^ mechanism. Based on the Wiberg bond index analysis, it is possible to conclude that all transition states are early in nature (δBav < 50%), and the corresponding values for compounds A to D turned out to be more synchronous than those for compounds E and F.

## Figures and Tables

**Table 1:** Thermodynamic properties at the wB97XD/6-311++g(2d,2p) level at 298.15 K, compared to experimental results mentioned by Katrice et al. [15].

Compound	Mechanism	Theory			Experimental		
		∆H^‡^	∆S^‡^	∆G^‡^	∆H^‡^	∆S^‡^	∆G^‡^
		kcal/mol	cal/molK	kcal/mol	kcal/mol	cal/molK	kcal/mol
A	S_N_^2^	11.45	−23.64	18.50	13.79	−18.24	19.22
B	S_N_^2^	11.50	−25.43	19.08	13.84	−19.98	19.79
	O-assistance	33.02	−2.17	33.67			
C	S_N_^2^	11.91	−23.67	18.97	15.25	−19.29	20.98
	O-assistance	26.60	−6.36	28.50			
D	S_N_^2^	13.44	−18.24	18.87	11.57	−23.80	18.64
E	S_N_^2^	16.89	−21.36	23.26	25.33	4.97	23.85
	N-assistance	41.38	−0.22	41.45			
F	S_N_^2^	17.66	−21.05	23.94	---	---	---
	N-assistance	23.45	−0.23	23.52	---	---	---

^‡^ Thermodynamic activation parameters.

**Table 2:** Values of work done associated with each region (reactant (R), transition state (TS), and product (P)) in the reaction force (RF) profiles, given in kcal/mol. Er—reaction energy.

Mechanism	Compound	W_1_	W_2_	W_3_	W_4_	Er
S_N_^2^	A	9.19	3.80	−16.01	−23.59	−26.61
	B	9.41	3.78	−16.36	−23.84	−27.01
	C	8.99	4.04	−15.21	−24.03	−26.21
	D	11.10	3.89	−17.03	−23.87	−25.91
	E	9.91	6.40	−18.05	−24.01	−25.75
	F	11.59	6.58	−15.70	−20.11	−17.64
N-assistance	F	11.89	8.72	−5.78	−9.67	5.16

**Table 3:** Geometric parameters for reactants (R), transition state (TS), and products (P) at the wB97XD/6-311++g(2d,2p) level.

**Mechanism**	**Compound**	**Stationary Point**	**S–C (Å)**	**C–Cl (Å)**	**Angle S–C–Cl**	**Imaginary Frequency (cm^−1^)**
S_N_^2^	A	R	3.680	1.807	174.43	−545.06
		TS	2.551	2.197		
		P	1.829	3.557		
	B	R	3.711	1.808	172.19	−543.78
		TS	2.554	2.187		
		P	1.829	3.421		
	C	R	3.642	1.811	172.06	−542.17
		TS	2.553	2.205		
		P	1.827	3.481		
	D	R	3.769	1.811	170.03	−530.77
		TS	2.565	2.208		
		P	1.831	3.614		
	E	R	3.372	1.822	163.54	−534.96
		TS	2.604	2.296		
		P	1.830	3.723		
	F	R	3.531	1.823	158.66	−525.35
		TS	2.612	2.326		
		P	1.849	3.119		
		**Stationary Point**	**N–C (Å)**	**C–Cl (Å)**	**Angle N–C–Cl**	**Imaginary Frequency (cm^−1^)**
N-assistance	F	R	2.384	1.815	155.99	−526.43
		TS	1.895	2.381		
		P	1.490	3.423		

**Table 4:** Charge changes, δQ*^x^* (*x* = S, C, or Cl), through the reaction pathway.

**Mechanims**	**Compound**	**δQ^S^**	**δQ^C^**	**δQ^Cl^**
S_N_^2^	A	0.363	0.043	−0.378
	B	0.374	0.092	−0.388
	C	0.361	0.097	−0.386
	D	0.382	0.108	−0.384
	E	0.44	0.161	−0.581
	F	0.316	0.164	−0.447
		**δQ^N^**	**δQ^C^**	**δQ^Cl^**
N-assistance	F	0.101	0.231	−0.486

**Table 5:** Wiberg bond indexes changes, average value, and synchronicity.

**Mechanism**	**Compound**	**δBi(S–C) %**	**δBi(C–Cl) %**	**δB_av_ %**	**Sy**
S_N_^2^	A	38.13	45.16	41.64	0.916
	B	39.97	46.55	43.26	0.924
	C	38.42	45.83	42.12	0.912
	D	37.22	45.87	41.55	0.896
	E	38.22	47.46	42.84	0.892
	F	39.92	49.93	44.93	0.889
		**δBi(N–C) %**	**δBi(C–Cl) %**	**δB_av_ %**	**Sy**
N-assistance	F	38.12	48.11	43.12	0.884

## Supplementary Materials

Supplementary materials can be found at http://www.mdpi.com/1422-0067/19/10/2864/s1.
