# Supplementary material for: New Insight into the Chloroacetanilide Herbicide Degradation Mechanism through a Nucleophilic Attack of Hydrogen Sulfide
Source: Int J Mol Sci. 2018 Sep 21;19(10):2864. doi: 10.3390/ijms19102864 (PMC6213056; doi:10.3390/ijms19102864)
Supplement: Supplementary file 1 [file ijms-19-02864-s001.pdf]

New Insight into the Chloroacetanilide Herbicide Degradation Mechanism through a Nucleophilic Attack of Hydrogen Sulfide

José R. Mora [1,2], Cristian Cervantes [1,2], Edgar Marquez [3,*]

[1]Universidad San Francisco de Quito, Instituto de Simulación Computacional (ISC-USFQ), Diego de Robles y Vía Interoceánica, Quito, Ecuador 17-1200-841.
[2]Universidad San Francisco de Quito, Grupo de Química Computacional y Teórica (QCT-USFQ), Departamento de Ingeniería Química, Diego de Robles y Vía Interoceánica, Quito, Ecuador 17-1200-841.
[3]Universidad del Norte, Departamento de Química y Biología, Facultad de Ciencias, Km 5, Vía Puerto Colombia, Barranquilla, Colombia.
* Correspondence: emarquezbrazon@gmail.com

Supporting Information

Table S1. Cartesian coordinates for all studied compounds considered in this study.

A_R

N	-0.04803900	-0.18970100	0.22815500
C	0.77843000	-0.35293200	-0.82020600
O	0.36902600	-0.35113500	-1.98562300
C	2.26000800	-0.57243700	-0.58154900
H	2.77825000	-0.41083200	-1.51805100
H	2.69014600	0.05384500	0.18988100
Cl	2.54236400	-2.29782700	-0.12214300
S	2.46404500	3.10020000	-0.48234900
H	1.39510400	2.34537600	-0.19310700
C	-1.44757700	-0.05760800	-0.06657600
C	-2.24830700	-1.18991900	-0.11776200
C	-1.98876300	1.19880000	-0.29923600
C	-3.60274600	-1.06201100	-0.39204400
H	-1.80887000	-2.16327200	0.05149400
C	-3.34315600	1.32225400	-0.57481700
H	-1.34709600	2.06925500	-0.26610700
C	-4.15152600	0.19326500	-0.61873900
H	-4.22741700	-1.94402000	-0.43043700
H	-3.76631900	2.30091000	-0.75584900
H	-5.20719300	0.29175800	-0.83265400
C	0.35963600	-0.17678900	1.65139500
H	1.44383200	-0.19845800	1.66214000
C	-0.14653700	-1.40942300	2.39029900
H	0.27065000	-1.41486800	3.39735500
H	-1.23347800	-1.40063300	2.47752400
H	0.15740700	-2.32600400	1.88598100
C	-0.08501200	1.10716600	2.33965500
H	-1.17102500	1.15992600	2.42165800
H	0.32915800	1.12766600	3.34757200
H	0.27036500	1.98682600	1.80321600

TS_A

N	0.01812000	0.02951100	0.23505600
C	0.89521100	-0.04675100	-0.78723500
O	0.51514000	-0.10478100	-1.96418000
C	2.36219800	-0.14092500	-0.50657600
H	2.99642200	-0.07299300	-1.36855400
H	2.80520300	-0.04123200	0.46149500
Cl	2.45458700	-2.33451700	-0.43703900
S	2.49176400	2.40692000	-0.51475100
H	1.87984300	2.53514400	0.66964000
C	-1.37563400	0.00059900	-0.10357000
C	-2.04971600	-1.21225500	-0.14063800
C	-2.04638300	1.18275300	-0.38764100
C	-3.40266400	-1.24053200	-0.45145500
H	-1.51245700	-2.12745500	0.06742000
C	-3.39768400	1.15115300	-0.69991600
H	-1.50674600	2.11965900	-0.36423100
C	-4.07831900	-0.05979400	-0.72906700
H	-3.92675600	-2.18622900	-0.47910700
H	-3.91892300	2.07280200	-0.92068400
H	-5.13227600	-0.08288800	-0.97104000
C	0.37678600	0.11951000	1.66564200
H	1.44047600	0.32540500	1.70400000
C	0.11272900	-1.19223600	2.39490300
H	0.47831500	-1.11508300	3.41926700
H	-0.95502200	-1.41181100	2.43537500
H	0.62473900	-2.01956700	1.90474200
C	-0.33134100	1.28574700	2.34436800
H	-1.40692400	1.11864400	2.40931700
H	0.05451900	1.38763300	3.35860600
H	-0.15566800	2.22209700	1.81459300

P_A

N	-0.05497300	0.22545900	0.25654100
C	0.79994800	0.31530200	-0.78197900
O	0.41057700	0.20513000	-1.95135300
C	2.25893300	0.61555300	-0.52142400
H	2.81077300	0.31857500	-1.40771700
H	2.67586500	0.08776000	0.32765800
Cl	2.46904400	-2.93071900	-0.35023600
S	2.53720700	2.41661300	-0.36595200
H	1.92104900	2.58648500	0.81316700
C	-1.44789600	0.07092200	-0.05591500
C	-2.02454800	-1.19018200	-0.09670300
C	-2.21370900	1.20040100	-0.31443800
C	-3.37712700	-1.31880400	-0.38525500
H	-1.41576400	-2.06405300	0.08980200
C	-3.56284100	1.06819400	-0.60627100
H	-1.74743200	2.17598100	-0.28716900
C	-4.14733300	-0.19192900	-0.63849500
H	-3.82632800	-2.30215600	-0.41592700

H	-4.15716500	1.94901300	-0.80802300
H	-5.20013800	-0.29498900	-0.86410700
C	0.32372500	0.29891500	1.68565000
H	1.37989700	0.54412800	1.71514100
C	0.14359700	-1.04563800	2.37868400
H	0.53250500	-0.97863200	3.39507300
H	-0.90950900	-1.32225200	2.44031300
H	0.68661300	-1.82845000	1.84805500
C	-0.42803500	1.41182200	2.40423200
H	-1.49445600	1.19478400	2.47224700
H	-0.03800300	1.50366100	3.41775000
H	-0.29937200	2.36908600	1.89817200

B_R

N	0.28617700	0.03723600	0.27998300
C	1.15681800	-0.03949700	-0.75537900
O	0.78017300	-0.19991300	-1.90998200
C	2.62739900	0.12116800	-0.40592100
H	2.87074800	1.17844100	-0.35948200
H	2.88145700	-0.34303700	0.54022900
Cl	3.68546900	-0.62128800	-1.64203400
C	-1.11366900	-0.16393500	0.01237400
C	-1.93072100	0.95125500	-0.20562300
C	-1.60481700	-1.46851700	-0.02025900
C	-3.27993300	0.72647800	-0.45462800
C	-2.96299500	-1.64970700	-0.27477200
C	-3.79224300	-0.56392700	-0.48977000
H	-3.94264300	1.56200000	-0.62889500
H	-3.36308600	-2.65536000	-0.30577300
H	-4.84432300	-0.71840600	-0.68986400
C	0.71382700	0.22610900	1.64994100
H	1.39873800	-0.57107100	1.95422000
C	-1.33564600	2.33977700	-0.16232300
H	-0.47633800	2.36704900	-0.83663800
H	-0.92722600	2.50778100	0.83646800
C	-2.28528100	3.47649200	-0.50982800
H	-1.75252200	4.42630200	-0.46657300
H	-2.69314100	3.36790500	-1.51593400
H	-3.12126600	3.53335100	0.18866500
C	-0.73224900	-2.66785000	0.25011800
H	-1.08990800	-3.49916300	-0.35823800
H	0.29515800	-2.47230800	-0.05520300
C	-0.75704600	-3.07779600	1.72385700
H	-1.77225700	-3.32293500	2.03947800
H	-0.12895400	-3.95371800	1.88905200
H	-0.39221800	-2.27365600	2.36412800
H	-0.17500900	0.17636600	2.27975900
O	1.34890200	1.47774200	1.77702100
C	2.21640200	1.52251900	2.89851500
H	2.65950500	2.51507800	2.91751200
H	1.66906500	1.35329900	3.82979300

H	3.00688400	0.77177500	2.80481200

B_TS

N	0.21663600	-0.00018900	0.28032500
C	1.15623300	0.12392800	-0.68068400
O	0.92444800	0.04999600	-1.87331500
C	2.52004900	0.42494500	-0.06789800
H	3.04867900	1.31131900	-0.36852200
H	2.99935400	-0.30540500	0.56116000
Cl	3.95197400	-0.70208800	-1.68472600
C	-1.16575200	-0.21555000	-0.02806500
C	-1.97988700	0.90072000	-0.25995000
C	-1.64254200	-1.52268000	-0.08499600
C	-3.31972200	0.66941300	-0.54518800
C	-2.99271700	-1.70908900	-0.37678900
C	-3.82137600	-0.62516900	-0.60227900
H	-3.98334000	1.50163400	-0.73089000
H	-3.38645100	-2.71629800	-0.42712200
H	-4.86724700	-0.78396700	-0.82946600
C	0.65104900	0.29376400	1.62016700
H	1.06002300	-0.58181900	2.13035300
C	-1.38844100	2.29057000	-0.19857200
H	-0.53388200	2.33158400	-0.87803900
H	-0.97697700	2.45127800	0.80094200
C	-2.34311400	3.42833500	-0.52756200
H	-1.81499300	4.37955200	-0.46548700
H	-2.74695500	3.33583600	-1.53667300
H	-3.18109600	3.46717700	0.16970000
C	-0.76306000	-2.71106900	0.20353600
H	-1.08543000	-3.54204400	-0.42458700
H	0.27045200	-2.49248000	-0.06333300
C	-0.83678700	-3.13178000	1.67254700
H	-1.85596800	-3.41181400	1.94317400
H	-0.18767700	-3.98749600	1.86032600
H	-0.52746800	-2.31918200	2.33133100
H	-0.15087100	0.74215100	2.20230300
O	1.69791100	1.23810500	1.39632600
C	2.57472700	1.42097600	2.52066200
H	3.32901900	2.13443700	2.20576800
H	1.98923500	1.82274100	3.34474600
H	3.03074600	0.47092000	2.79872600

B_P

N	0.07701800	0.04877700	0.39016200
C	1.01114000	0.29980300	-0.55099300
O	0.87031300	0.17767900	-1.75078900
C	2.24254200	0.81159800	0.15921100
H	2.65997000	1.70149400	-0.29568400
H	2.98735100	0.03216700	0.31054300
Cl	4.29177700	-0.88207700	-1.79823000
C	-1.29862000	-0.19429600	0.07810500

C	-2.11128700	0.91685100	-0.18059200
C	-1.75451900	-1.50662900	0.00522000
C	-3.44073200	0.67340000	-0.49861700
C	-3.09589200	-1.70436200	-0.31784700
C	-3.92782800	-0.62679200	-0.56299300
H	-4.10622600	1.49896200	-0.70619100
H	-3.47902600	-2.71469700	-0.38269200
H	-4.96606700	-0.79563500	-0.81626900
C	0.54215200	0.29504500	1.72117700
H	0.94923900	-0.58769100	2.21377800
C	-1.52340700	2.30895800	-0.12353000
H	-0.67157100	2.35180000	-0.80618400
H	-1.11120500	2.47549500	0.87479200
C	-2.47812200	3.44300800	-0.46200000
H	-1.95189300	4.39517600	-0.39940700
H	-2.87373600	3.34637800	-1.47391100
H	-3.32135400	3.48196400	0.22882800
C	-0.85738300	-2.68216100	0.29034000
H	-1.15624300	-3.50985100	-0.35340400
H	0.17369400	-2.43686800	0.03631400
C	-0.93545000	-3.12696800	1.75162100
H	-1.95004100	-3.43571900	2.00775200
H	-0.26764900	-3.96971100	1.93190900
H	-0.65248600	-2.31837400	2.42699900
H	-0.17948700	0.82321000	2.33571800
O	1.66644500	1.20927800	1.45699300
C	2.61400000	1.37158600	2.56380600
H	3.31379700	2.13182300	2.23703800
H	2.02271900	1.70765300	3.40842300
H	3.09639900	0.41539400	2.74702800

C_R

N	0.16117300	0.04237600	0.18120800
C	-0.14541200	0.99284700	-0.72362600
O	0.54418800	1.19954500	-1.72393700
C	-1.35174800	1.88075100	-0.48040900
H	-1.64649800	2.32716700	-1.42066100
H	-2.20043400	1.39519300	-0.01597500
Cl	-0.84722400	3.23296400	0.61268600
S	-3.10890600	-0.22392700	-2.87780000
H	-3.49287100	0.13417800	-1.64499300
C	1.40466100	-0.65911700	-0.00313400
C	2.56821500	-0.10896100	0.54446400
C	1.42505200	-1.85582000	-0.72445300
C	3.76121600	-0.80987700	0.39449500
C	2.64059100	-2.52218100	-0.85555500
C	3.79922900	-2.01119100	-0.29350500
H	4.66796300	-0.40060100	0.82235300
H	2.67290200	-3.44972900	-1.41285100
H	4.73405300	-2.54523600	-0.40102900
C	-0.69818600	-0.26258700	1.34911200

H	-1.11791900	0.67975600	1.69712200
C	2.58852200	1.22546900	1.25005800
H	3.21424800	1.13640600	2.13960400
H	1.59148800	1.49675600	1.59102000
C	3.13443200	2.34583100	0.36249200
H	3.14857900	3.29001300	0.90835300
H	4.15308500	2.12656200	0.03881500
H	2.51713300	2.47640500	-0.52641700
C	0.19190900	-2.43407700	-1.36040300
H	-0.31449300	-3.11718200	-0.67630400
H	-0.52484900	-1.66719400	-1.65276100
C	0.07965800	-0.86927500	2.50586200
H	-0.61734400	-1.05103400	3.32289800
H	0.53045000	-1.82149400	2.22734400
H	0.85755300	-0.20240600	2.87158100
C	-1.86054100	-1.16849600	0.94167500
H	-1.53178900	-2.21233300	0.90693800
H	-2.23492200	-0.90022300	-0.05148500
O	-2.88904700	-1.02279500	1.90186800
C	-3.98904300	-1.86600200	1.61466600
H	-3.69037000	-2.91864100	1.63197400
H	-4.74227300	-1.69344700	2.38036500
H	-4.41203600	-1.63370000	0.63198400
H	0.46311800	-3.00491800	-2.24752600

C_TS

N	0.05543400	0.02845900	0.04871000
C	-0.26911100	0.92963300	-0.90567000
O	0.48847900	1.17807900	-1.84993500
C	-1.55463900	1.69222500	-0.79526500
H	-1.73469500	2.41252100	-1.56554800
H	-2.36527500	1.39649800	-0.15893400
Cl	-0.83095300	3.23821500	0.60069200
S	-2.64192600	0.13564700	-2.50323400
H	-3.70489100	-0.09114700	-1.72009300
C	1.32770300	-0.63206000	-0.08514200
C	2.47229100	-0.02431500	0.44194600
C	1.39163200	-1.86584600	-0.74092000
C	3.68473500	-0.70166800	0.34306200
C	2.62512400	-2.50574300	-0.82603600
C	3.76364800	-1.93588400	-0.27942600
H	4.57547800	-0.24664200	0.75835500
H	2.68747300	-3.46047700	-1.33271200
H	4.71335300	-2.44983200	-0.34805000
C	-0.79031900	-0.23490100	1.23430500
H	-1.26070800	0.70998500	1.49986300
C	2.45305800	1.34319400	1.08044100
H	2.97546600	1.28648400	2.03747800
H	1.43199500	1.65313600	1.29011000
C	3.12099900	2.40552300	0.20611200
H	3.09406500	3.37682200	0.70177600

H	4.16466800	2.15675900	0.00805800
H	2.60721000	2.50001100	-0.75099600
C	0.18005200	-2.51200200	-1.35276400
H	-0.31694300	-3.16524900	-0.63328700
H	-0.55079200	-1.77879900	-1.69220700
C	0.01620300	-0.69503400	2.43916200
H	-0.67405100	-0.85359100	3.26698300
H	0.52865800	-1.63652500	2.24187600
H	0.74752700	0.04748800	2.75110500
C	-1.90105100	-1.23372900	0.91432400
H	-1.52427500	-2.25995500	0.98416700
H	-2.27755200	-1.07378200	-0.09897200
O	-2.94473600	-1.04419000	1.85339700
C	-4.02127000	-1.93113100	1.61679800
H	-3.69966800	-2.97303700	1.71174400
H	-4.78734300	-1.72316900	2.36084000
H	-4.43878800	-1.77950400	0.61608000
H	0.47379700	-3.12792800	-2.20189000

C_P

N	0.03038200	-0.14250200	-0.02502200
C	-0.33960100	0.60826500	-1.08612600
O	0.44252300	0.83839600	-2.01371000
C	-1.75323500	1.15071300	-1.17097900
H	-1.67796500	2.20925300	-1.40558600
H	-2.33979100	1.05340200	-0.26793800
Cl	-0.75445400	3.59576400	1.09574000
S	-2.67348300	0.41507900	-2.56769600
H	-2.76816500	-0.82958400	-2.07823300
C	1.33803500	-0.74386100	-0.08171900
C	2.44258100	-0.05669500	0.42569700
C	1.46529000	-2.01064600	-0.66426300
C	3.68292500	-0.69182100	0.39689500
C	2.72293600	-2.60372400	-0.68521000
C	3.82457800	-1.95654300	-0.14576400
H	4.54558700	-0.17717000	0.80153200
H	2.83581500	-3.58268000	-1.13319500
H	4.79468700	-2.43549100	-0.16103100
C	-0.81345600	-0.31924000	1.18086100
H	-1.29671700	0.63875200	1.36743200
C	2.35336700	1.34962500	0.96486900
H	2.80320000	1.37089400	1.95998500
H	1.31524400	1.65795900	1.07633200
C	3.06947500	2.36114200	0.06887100
H	2.98734100	3.36424800	0.48946000
H	4.12942800	2.12425000	-0.03366900
H	2.62725000	2.37573100	-0.92829100
C	0.29198700	-2.73132800	-1.26843200
H	-0.32567700	-3.19680000	-0.49966600
H	-0.34831400	-2.06112400	-1.84114100
C	0.00040900	-0.66426600	2.41873200

H	-0.68919100	-0.77260400	3.25500600
H	0.53562000	-1.60586000	2.29731700
H	0.71156500	0.11840500	2.67236500
C	-1.90212100	-1.36836600	0.95548900
H	-1.49832100	-2.37556200	1.10393100
H	-2.29752700	-1.31691800	-0.06293500
O	-2.94157900	-1.12624300	1.88171100
C	-3.99050400	-2.06712900	1.74949500
H	-3.63158100	-3.08352400	1.93810200
H	-4.75186100	-1.81249800	2.48348000
H	-4.42709500	-2.02523500	0.74662900
H	0.63907600	-3.51992500	-1.93394400

D_R

N	-0.09081700	-0.18907600	0.31158000
C	0.75311500	-0.36653000	-0.70234900
C	2.22444400	-0.53642200	-0.40207600
H	2.78822400	-0.45334100	-1.31987200
H	2.60538300	0.17564000	0.32282800
Cl	2.54640400	-2.20080000	0.23535900
S	2.61185500	3.20393800	-0.15164200
H	1.55310400	2.38186000	-0.15698400
C	-1.49848500	-0.07515300	0.03704900
C	-2.28372700	-1.21773400	0.02296500
C	-2.05639700	1.17296400	-0.19351300
C	-3.64408200	-1.10895000	-0.22658800
H	-1.82884200	-2.18330400	0.19500800
C	-3.41710600	1.27614200	-0.44294500
H	-1.42533800	2.05121100	-0.18607700
C	-4.21183700	0.13674100	-0.45993400
H	-4.25886000	-1.99854700	-0.23962000
H	-3.85588700	2.24763500	-0.62508400
H	-5.27247700	0.21992100	-0.65470600
C	0.30202500	-0.11871100	1.74626900
H	1.38364900	-0.16354900	1.77451100
C	-0.23900500	-1.30815600	2.52882900
H	0.18139800	-1.28098000	3.53408600
H	-1.32457600	-1.26726900	2.61735800
H	0.04173300	-2.25203200	2.06351200
C	-0.12348300	1.20413400	2.36856700
H	-1.20763300	1.27966300	2.45063000
H	0.29476700	1.26242900	3.37337700
H	0.24971000	2.04999500	1.79163800
S	0.29092700	-0.42121100	-2.31331900

D_TS

N	-0.01723200	0.01549300	0.32543900
C	0.88620300	-0.06550200	-0.65294100
C	2.33358300	-0.11830000	-0.30421800
H	3.01617400	-0.07741800	-1.12842100
H	2.72344500	0.04130700	0.67798800

Cl	2.52657700	-2.30390300	-0.05469000
S	2.46026600	2.44344100	-0.31977600
H	2.20172300	2.51351700	0.99312800
C	-1.41620200	-0.00722500	-0.00357600
C	-2.09591800	-1.21583100	-0.01967500
C	-2.08263700	1.18041500	-0.26835800
C	-3.45383500	-1.23575900	-0.30638100
H	-1.56055500	-2.13383700	0.17947600
C	-3.43924600	1.15613500	-0.55536500
H	-1.53686400	2.11360600	-0.25692600
C	-4.12691100	-0.05116700	-0.57430900
H	-3.98433900	-2.17811300	-0.32122100
H	-3.95936500	2.08117700	-0.76405400
H	-5.18521100	-0.06807400	-0.79702400
C	0.30462900	0.13166400	1.77105900
H	1.37416100	0.27677700	1.84110000
C	-0.05125200	-1.14432200	2.52335000
H	0.31165000	-1.06087600	3.54802800
H	-1.13008600	-1.29690400	2.56081500
H	0.41525000	-2.01301900	2.06070900
C	-0.35493800	1.35469900	2.39535700
H	-1.43784900	1.24558900	2.45236800
H	0.02542500	1.46879700	3.41049600
H	-0.12018500	2.26057200	1.83740600
S	0.47689500	-0.19494400	-2.28471600

D_P

N	-0.10395500	0.22330700	0.30636600
C	0.75714400	0.29655500	-0.70812900
C	2.20829100	0.58569700	-0.40656000
H	2.79487400	0.32347100	-1.27939600
H	2.59361600	0.01738400	0.43092400
Cl	2.67010900	-2.94067500	0.23351200
S	2.51641500	2.37573200	-0.17383000
H	1.98245000	2.49637900	1.04978100
C	-1.50733400	0.06988900	0.03007600
C	-2.08629200	-1.18913000	0.01966600
C	-2.27360400	1.20326400	-0.20094100
C	-3.44713800	-1.31277700	-0.22735400
H	-1.47522200	-2.06433000	0.19043700
C	-3.63110800	1.07487100	-0.44929800
H	-1.80157500	2.17616200	-0.19433400
C	-4.22002200	-0.18366400	-0.46249900
H	-3.90157700	-2.29403500	-0.23859200
H	-4.22792100	1.95785300	-0.63322200
H	-5.27947600	-0.28298400	-0.65618100
C	0.26517500	0.31896700	1.74606400
H	1.32834900	0.52188500	1.78354000
C	0.03046900	-1.00287500	2.46523700
H	0.42144100	-0.92043200	3.47970800
H	-1.03139400	-1.23912200	2.53378700

H	0.54985700	-1.81705000	1.95926000
C	-0.45473300	1.47285800	2.43142100
H	-1.52478800	1.28405700	2.51634400
H	-0.05096200	1.58239100	3.43774700
H	-0.30741200	2.41199700	1.89809200
S	0.30996500	0.11423100	-2.31931400

E_R

N	0.62243500	-0.12544000	0.65217300
C	-0.52701800	-0.41570700	0.00031300
O	-0.51297700	-0.83971000	-1.16026400
C	-3.07692500	-0.37681200	-0.09479900
H	-3.87691200	0.25332100	0.27254100
H	-2.90642300	-0.16572600	-1.14147100
Cl	-3.75470300	-2.06632000	-0.02505800
S	-2.41677800	2.75415400	-1.15983300
H	-1.23210900	2.18990300	-0.88616100
C	1.85236100	-0.23708000	-0.08013800
C	2.30840400	0.83174400	-0.84092000
C	2.59413400	-1.40859500	-0.00900800
C	3.51493300	0.73041100	-1.52092200
H	1.71437600	1.73367300	-0.90273400
C	3.79862500	-1.50759600	-0.69080400
H	2.22746700	-2.23601500	0.58267500
C	4.26255700	-0.43741000	-1.44502600
H	3.86820300	1.56438500	-2.11212500
H	4.37446600	-2.42122600	-0.63185200
H	5.20238400	-0.51469700	-1.97476900
C	0.67703100	0.48408400	2.00153000
H	-0.25534900	0.22522600	2.49225100
C	0.78102700	2.00287100	1.92433600
H	0.70704600	2.42450900	2.92717000
H	1.73775100	2.30946800	1.49946900
H	-0.02079300	2.42031500	1.31363600
C	1.80183500	-0.11151000	2.83686000
H	2.78281000	0.15778900	2.44438400
H	1.72695500	0.28068900	3.85096400
H	1.73054400	-1.19793900	2.88361800
C	-1.83150500	-0.19520000	0.74618100
H	-1.84282500	0.83318800	1.10736800
H	-1.86091900	-0.83783700	1.62662800

E_TS

N	0.58430200	-0.13703900	0.60954300
C	-0.56013400	-0.36600600	-0.07003600
O	-0.54339100	-0.82234600	-1.21753900
C	-3.03251100	-0.00308200	-0.31061600
H	-3.95129500	0.42555800	0.03894400
H	-2.91358600	-0.19549300	-1.35600100
Cl	-3.93063800	-2.10348300	-0.07831400
S	-2.53627700	2.42689400	-1.10396600

H	-1.20921100	2.26671300	-1.00261200
C	1.81517600	-0.26535900	-0.11699600
C	2.27026300	0.79947800	-0.88381700
C	2.54917200	-1.44101300	-0.04771600
C	3.46989600	0.69092900	-1.57314700
H	1.67883200	1.70342100	-0.94254300
C	3.74831000	-1.54667500	-0.73844300
H	2.18157800	-2.26591400	0.54677000
C	4.21161900	-0.48089700	-1.49914600
H	3.82286800	1.52135100	-2.16948200
H	4.32019200	-2.46294100	-0.68229100
H	5.14692200	-0.56474400	-2.03583200
C	0.63225300	0.45807700	1.96515800
H	-0.29975600	0.18485300	2.45070300
C	0.73610600	1.97727000	1.90322200
H	0.66077500	2.39084600	2.90923300
H	1.69442700	2.28478700	1.48243800
H	-0.06205500	2.40300900	1.29377700
C	1.75679800	-0.14409600	2.79562800
H	2.73772800	0.13088300	2.40717700
H	1.67926000	0.23856400	3.81311100
H	1.68720400	-1.23093600	2.83220000
C	-1.87214600	-0.07311100	0.63138500
H	-1.82014700	0.86220400	1.18056700
H	-2.04426700	-0.85830800	1.36929100

E_P

N	0.67494500	-0.01043300	0.65716300
C	-0.47378000	-0.11834100	-0.04558100
O	-0.46671600	-0.52872200	-1.21357500
C	-2.79696800	0.79092200	-0.37841500
H	-3.74707800	0.95464100	0.12604600
H	-2.97259200	0.10337300	-1.19924200
Cl	-4.17581100	-2.64186800	0.04216100
S	-2.34503700	2.42893300	-1.05757300
H	-1.25305700	2.03358700	-1.72602000
C	1.90028400	-0.18944400	-0.06902500
C	2.41126800	0.86631100	-0.81325300
C	2.57323900	-1.40209100	-0.02395900
C	3.60383600	0.71088100	-1.50534000
H	1.86760500	1.80066700	-0.85228800
C	3.76752700	-1.55377000	-0.71492300
H	2.16297700	-2.21994000	0.55195700
C	4.28528000	-0.49833200	-1.45439200
H	3.99912600	1.53414400	-2.08484100
H	4.29244000	-2.49862200	-0.67616600
H	5.21595600	-0.61895500	-1.99214000
C	0.75485300	0.52732600	2.03633100
H	-0.17932800	0.26063800	2.52106900
C	0.90603000	2.04338400	2.02960400
H	0.87305700	2.42145500	3.05177000

H	1.86148100	2.33538000	1.59130400
H	0.10512300	2.51569000	1.45974500
C	1.86808600	-0.13926200	2.83190200
H	2.85404100	0.12178000	2.44672900
H	1.80824000	0.20655100	3.86365100
H	1.76709400	-1.22415300	2.82848500
C	-1.79038300	0.23744900	0.61453800
H	-1.66573100	0.94157100	1.43070600
H	-2.19670300	-0.69051500	1.02906100

F_R

N	-0.15246100	0.65053800	0.04993000
C	-2.13092900	-0.55189900	-0.51839500
H	-2.54048400	0.29983200	-1.05094400
H	-1.56677800	-1.17800500	-1.20295700
Cl	-3.55660200	-1.54419900	0.00730300
C	1.09410300	-0.05027600	0.04964900
C	1.59059500	-0.52384100	-1.15890400
C	1.82817500	-0.26598400	1.21573700
C	2.80717800	-1.19092100	-1.21348800
H	1.01776000	-0.35452200	-2.06058800
C	3.04578100	-0.93113200	1.16261200
H	1.44503800	0.09553800	2.16180900
C	3.53996000	-1.39277400	-0.05255900
H	3.18181800	-1.54920000	-2.16314100
H	3.61202600	-1.08897500	2.07115900
H	4.48996500	-1.90872700	-0.09151700
C	-1.25498400	-0.10246400	0.63875600
H	-1.82443500	0.50133700	1.34494600
H	-0.88586200	-0.97073500	1.18620500
C	-0.08394000	2.09179700	0.33913700
H	0.12764600	2.25786900	1.40606300
C	1.02944600	2.73543800	-0.48078200
H	1.03549500	3.81144600	-0.30544600
H	2.01393400	2.34868400	-0.22072300
H	0.86339300	2.56239400	-1.54637500
C	-1.40645200	2.77266600	0.00085800
H	-2.24514800	2.38040300	0.57442100
H	-1.33049100	3.83816600	0.21951000
H	-1.63166000	2.65623100	-1.06130100

F_TS

N	-0.21401100	0.54891500	0.15028800
C	-1.76503900	-0.39217600	-0.39596400
H	-2.33519300	0.37102400	-0.89390600
H	-1.32408900	-1.17881000	-0.98373900
Cl	-3.73780100	-1.69157900	-0.09945300
C	1.04157700	-0.14986000	0.07103300
C	1.51050900	-0.58899400	-1.15707700
C	1.78823600	-0.35862300	1.22465100
C	2.73568200	-1.23841500	-1.23384900

H	0.91779600	-0.41920500	-2.04542600
C	3.01243800	-1.00435400	1.14350500
H	1.41036900	-0.01290800	2.17812000
C	3.48801500	-1.44514300	-0.08594400
H	3.10006100	-1.58279300	-2.19204700
H	3.59473700	-1.16476300	2.04068700
H	4.44182500	-1.95125700	-0.14727600
C	-1.24158700	-0.13831400	0.94035600
H	-1.82136600	0.51986100	1.57568000
H	-0.87036400	-0.99953000	1.48233200
C	-0.13801000	2.03312600	0.29275500
H	0.11190300	2.22837600	1.34056100
C	0.95684700	2.60633900	-0.59347400
H	0.95955700	3.69019600	-0.48337600
H	1.94511000	2.23911800	-0.32314700
H	0.77046300	2.37005300	-1.64248700
C	-1.46783500	2.69961100	-0.02836700
H	-2.29390500	2.32130000	0.57204400
H	-1.37671500	3.76508200	0.17924900
H	-1.71449200	2.57951100	-1.08432500

F_P

N	-0.11198900	0.63914000	0.16458300
C	-1.28658800	0.02477200	-0.51672400
H	-1.96638100	0.73100900	-0.96256800
H	-1.05032500	-0.84930400	-1.10148800
Cl	-4.10810000	-1.86923200	-0.10921200
C	1.16008100	-0.06983600	0.08158600
C	1.62549900	-0.50808500	-1.14447800
C	1.88931400	-0.24731900	1.24430300
C	2.85444100	-1.14928000	-1.20381300
H	1.04297500	-0.34589800	-2.04063400
C	3.11764900	-0.88664600	1.17407000
H	1.49851700	0.10467800	2.18955100
C	3.59959900	-1.33820900	-0.04796100
H	3.22776200	-1.49870700	-2.15617200
H	3.69436800	-1.03584700	2.07612400
H	4.55600600	-1.84003800	-0.09856100
C	-1.17856900	-0.05079900	0.94217500
H	-1.77937600	0.59400500	1.56227800
H	-0.85105600	-0.97594000	1.38978100
C	0.02853100	2.15030100	0.26561900
H	0.47965600	2.29768200	1.24600300
C	0.96163600	2.65019500	-0.82316500
H	0.99818500	3.73685600	-0.76486800
H	1.97514200	2.27196100	-0.71066500
H	0.58474800	2.37355300	-1.80912900
C	-1.30191900	2.87946800	0.20522700
H	-2.03976300	2.50854000	0.91304300
H	-1.10394400	3.91896800	0.46207600
H	-1.72249400	2.86250100	-0.79983500

Table S2. Net atomic charges for all substrates under study.

Compound		S	C	Cl
	R	-1.018	-0.408	-0.107
A	TS	-0.655	-0.365	-0.485
	P	-0.045	-0.564	-0.982
	R	-1.030	-0.416	-0.101
B	TS	-0.656	-0.324	-0.489
	P	-0.056	-0.564	-0.974
	R	-1.009	-0.408	-0.101
C	TS	-0.648	-0.311	-0.487
	P	-0.025	-0.575	-0.973
	R	-1.019	-0.401	-0.100
D	TS	-0.637	-0.293	-0.484
	P	-0.032	-0.561	-0.981
	R	-1.016	-0.322	-0.144
E	TS	-0.576	-0.161	-0.725
	P	-0.103	-0.473	-0.989
	R	-1.076	-0.322	-0.141
F	TS	-0.760	-0.158	-0.588
	P	-0.124	-0.497	-0.974
		N	C	Cl
	R	-0.605	-0.336	-0.095
F	TS	-0.504	-0.105	-0.581
	P	-0.373	-0.136	-0.975
